# Bis[μ-3-ethyl-4-phenyl-5-(2-pyrid­yl)-4*H*-1,2,4-triazole]bis­[dichloridocopper(II)]

**DOI:** 10.1107/S160053680804035X

**Published:** 2008-12-06

**Authors:** Zuoxiang Wang, Chunyi Liu, Xiaoming Zhang, Xiaoning Gong

**Affiliations:** a Ordered Matter Science Research Center, Southeast University, Nanjing 210096, People’s Republic of China

## Abstract

The asymmetric unit of the title compound, [Cu_2_Cl_4_(C_15_H_14_N_4_)_2_], contains two halves of two centrosymmetric dinuclear mol­ecules, *A* and *B*. The conformations of the two crystallographically independent mol­ecules are slightly different: in *A*, the Cu⋯Cu separation is 4.174 (9) Å and the dihedral angle between the triazole and phenyl rings is 74.23 (11)°; these values are 4.137 (9) Å and 68.58 (13)°, respectively, in *B*. In each mol­ecule, the copper(II) ions have a distorted trigonal–bipyramidal coordination geometry with a CuCl_2_NN′N′′ chromophore. The crystal packing exhibits weak inter­molecular C—H⋯Cl inter­actions.

## Related literature

For the magnetic and spin-crossover properties of 1,2,4-triazole complexes, see: Kahn & Martinez (1998 ▶); Klingele *et al.* (2005 ▶); Matouzenko *et al.* (2004 ▶); Moliner *et al.* (2001 ▶); For the fluorescent properties of 1,2,4-triazole complexes, see: Chen *et al.* (2008 ▶); Matsukizono *et al.* (2008 ▶).
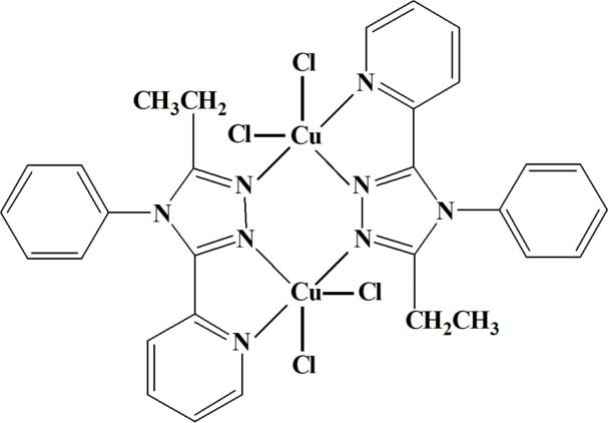


## Experimental

### 

#### Crystal data

[Cu_2_Cl_4_(C_15_H_14_N_4_)_2_]
*M* *_r_* = 769.48
Triclinic, 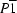

*a* = 9.3395 (11) Å
*b* = 12.8096 (14) Å
*c* = 13.9234 (16) Å
α = 92.533 (2)°
β = 94.596 (2)°
γ = 90.452 (2)°
*V* = 1658.6 (3) Å^3^
*Z* = 2
Mo *K*α radiation
μ = 1.64 mm^−1^
*T* = 273 (2) K
0.30 × 0.26 × 0.24 mm

#### Data collection

Bruker SMART APEX CCD area-detector diffractometer
Absorption correction: multi-scan (*SADABS*; Bruker, 2000 ▶) *T*_min_ = 0.62, *T*_max_ = 0.68
8289 measured reflections
5716 independent reflections
4065 reflections with *I* > 2σ(*I*)
*R*_int_ = 0.032

#### Refinement

*R*[*F* ^2^ > 2σ(*F* ^2^)] = 0.044
*wR*(*F* ^2^) = 0.069
*S* = 1.05
5716 reflections
399 parameters
H-atom parameters constrained
Δρ_max_ = 0.55 e Å^−3^
Δρ_min_ = −0.40 e Å^−3^

### 

Data collection: *SMART* (Bruker, 2000 ▶); cell refinement: *SAINT* (Bruker, 2000 ▶); data reduction: *SAINT*; program(s) used to solve structure: *SHELXTL* (Sheldrick, 2008 ▶); program(s) used to refine structure: *SHELXTL*; molecular graphics: *SHELXTL*; software used to prepare material for publication: *SHELXTL*.

## Supplementary Material

Crystal structure: contains datablocks I, global. DOI: 10.1107/S160053680804035X/cv2472sup1.cif

Structure factors: contains datablocks I. DOI: 10.1107/S160053680804035X/cv2472Isup2.hkl

Additional supplementary materials:  crystallographic information; 3D view; checkCIF report

## Figures and Tables

**Table 1:** Hydrogen-bond geometry (Å, °)

*D*—H⋯*A*	*D*—H	H⋯*A*	*D*⋯*A*	*D*—H⋯*A*
C4—H4⋯Cl2^i^	0.93	2.78	3.445 (4)	129
C15—H15⋯Cl3^ii^	0.93	2.78	3.579 (4)	145
C16—H16⋯Cl1^iii^	0.93	2.76	3.518 (4)	139
C19—H19⋯Cl4^iv^	0.93	2.69	3.365 (4)	130

Symmetry codes: (i) 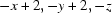; (ii) 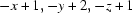; (iii) 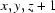; (iv) 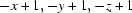.

**Fig. 1.**
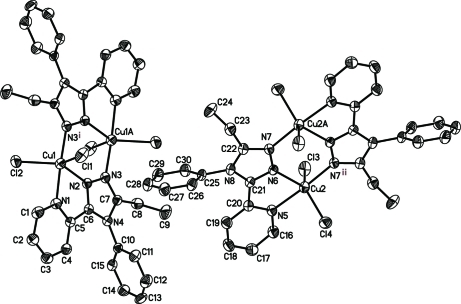
View of (I) with the atomic labelling and 30% probability displacement ellipsoids [symmetry codes: (i) -x + 1,-y + 2,-z; (ii) -x,-y + 1,-z + 1.

## supplementary crystallographic information

### Comment

As the 1,2,4-triazole ring posesses strong electron donors and coordination capability to transition metal ions, the coordination chemistry of 1,2,4-triazole derivatives has gained great attention in recent years (Klingele *et al.*, 2005; Chen *et al.*, 2008; Matsukizono *et al.*, 2008). Some complexes of 1,2,4-triazoles with iron(II) have spin-crossover properties, which can be used as molecular-based memory devices, displays and optical switches (Kahn & Martinez, 1998; Moliner *et al.*, 2001; Matouzenko *et al.*, 2004). We report here the crystal structure analysis of the title compound, (I) (Fig. 1).

The asymmetric unit of the title compound contains two halves of two centrosymmetric dinuclear molecules, A and B, respectively. In A, the dihedral angle between the triazole and pyridine rings is 11.21 (16)°, and that between the triazole and benzene rings is 74.22 (11)°; those values in B are 9.02 (16)° and 68.58 (13)°, respectively.

The crystal packing exhibits weak intermolecular C—H···Cl interactions (Table 1).

### Experimental

The title compound was prepared by reaction of 3-ethyl-4-phenyl-5-(2-pyridyl)-1,2,4-triazole with copper(II) chloride in ethanol and water. To a warm solution of 0.501 grams of 3-ethyl-4-phenyl-5-(2-pyridyl)-1,2,4-triazole (2.0 mmol) in 10 ml e thanol, 0.682 grams of copper(II) chloride dihydrate (4.0 mmol) in 10 ml water was added. The filtrate was left to stand at room temperature for several days, and single crystals suitable for X-ray diffraction were collected.

### Refinement

All H atoms were first located in a difference Fourier map, but placed in idealized positions (C—H = 0.93 (aromatic), 0.96 (methyl) or 0.97 Å (methylene)), and allowed to ride on their parent atoms with U_iso_(H) values of 1.2 or 1.5 times U_eq_(C).

### Crystal data

**Table:**

[Cu_2_Cl_4_(C_15_H_14_N_4_)_2_]	*Z* = 2
*M**_r_* = 769.48	*F*(000) = 780
Triclinic, *P*1	*D*_x_ = 1.541 Mg m^−^^3^
Hall symbol: -P 1	Mo *K*α radiation, λ = 0.71073 Å
*a* = 9.3395 (11) Å	Cell parameters from 2456 reflections
*b* = 12.8096 (14) Å	θ = 2.2–24.2°
*c* = 13.9234 (16) Å	µ = 1.64 mm^−^^1^
α = 92.533 (2)°	*T* = 273 K
β = 94.596 (2)°	Block, green
γ = 90.452 (2)°	0.30 × 0.26 × 0.24 mm
*V* = 1658.6 (3) Å^3^	

### Data collection

**Table:**

Bruker SMART APEX CCD area-detector diffractometer	5716 independent reflections
Radiation source: sealed tube	4065 reflections with *I* > 2σ(*I*)
graphite	*R*_int_ = 0.032
φ and ω scans	θ_max_ = 25.0°, θ_min_ = 2.1°
Absorption correction: multi-scan (*SADABS*; Bruker, 2000)	*h* = −9→11
*T*_min_ = 0.62, *T*_max_ = 0.68	*k* = −15→10
8289 measured reflections	*l* = −16→16

### Refinement

**Table:**

Refinement on *F*^2^	Primary atom site location: structure-invariant direct methods
Least-squares matrix: full	Secondary atom site location: difference Fourier map
*R*[*F*^2^ > 2σ(*F*^2^)] = 0.044	Hydrogen site location: inferred from neighbouring sites
*wR*(*F*^2^) = 0.069	H-atom parameters constrained
*S* = 1.05	*w* = 1/[σ^2^(*F*_o_^2^) + (0.01*P*)^2^] where *P* = (*F*_o_^2^ + 2*F*_c_^2^)/3
5716 reflections	(Δ/σ)_max_ = 0.004
399 parameters	Δρ_max_ = 0.55 e Å^−^^3^
0 restraints	Δρ_min_ = −0.40 e Å^−^^3^

### Special details

**Table:**

Geometry. All e.s.d.'s (except the e.s.d. in the dihedral angle between two l.s. planes) are estimated using the full covariance matrix. The cell e.s.d.'s are taken into account individually in the estimation of e.s.d.'s in distances, angles and torsion angles; correlations between e.s.d.'s in cell parameters are only used when they are defined by crystal symmetry. An approximate (isotropic) treatment of cell e.s.d.'s is used for estimating e.s.d.'s involving l.s. planes.
Refinement. Refinement of *F*^2^ against ALL reflections. The weighted *R*-factor *wR* and goodness of fit *S* are based on *F*^2^, conventional *R*-factors *R* are based on *F*, with *F* set to zero for negative *F*^2^. The threshold expression of *F*^2^ > σ(*F*^2^) is used only for calculating *R*-factors(gt) *etc*. and is not relevant to the choice of reflections for refinement. *R*-factors based on *F*^2^ are statistically about twice as large as those based on *F*, and *R*- factors based on ALL data will be even larger.

### Fractional atomic coordinates and isotropic or equivalent isotropic displacement parameters (Å^2^)

**Table:**

	*x*	*y*	*z*	*U*_iso_*/*U*_eq_	
Cu1	0.61708 (5)	0.91845 (3)	−0.09989 (3)	0.04319 (14)	
Cu2	0.14043 (5)	0.57176 (3)	0.60559 (3)	0.04375 (14)	
Cl1	0.52503 (13)	0.75357 (7)	−0.12247 (7)	0.0706 (4)	
Cl2	0.71352 (11)	1.02338 (8)	−0.20518 (8)	0.0645 (3)	
Cl3	0.05265 (12)	0.73248 (7)	0.64432 (7)	0.0655 (3)	
Cl4	0.25757 (11)	0.44618 (8)	0.68906 (8)	0.0663 (3)	
N1	0.8159 (3)	0.8654 (2)	−0.0588 (2)	0.0443 (8)	
N2	0.6455 (3)	0.9681 (2)	0.05218 (19)	0.0379 (7)	
N3	0.5746 (3)	1.0170 (2)	0.1247 (2)	0.0402 (8)	
N4	0.7742 (3)	0.9542 (2)	0.1897 (2)	0.0377 (7)	
N5	0.3290 (3)	0.6315 (2)	0.5707 (2)	0.0428 (8)	
N6	0.1338 (3)	0.5377 (2)	0.45041 (19)	0.0381 (7)	
N7	0.0455 (3)	0.4958 (2)	0.3749 (2)	0.0388 (8)	
N8	0.2338 (3)	0.5611 (2)	0.3164 (2)	0.0401 (8)	
C1	0.9021 (5)	0.8246 (3)	−0.1216 (3)	0.0562 (11)	
H1	0.8662	0.8134	−0.1855	0.067*	
C2	1.0420 (4)	0.7983 (3)	−0.0963 (3)	0.0551 (11)	
H2	1.0988	0.7685	−0.1419	0.066*	
C3	1.0958 (4)	0.8167 (3)	−0.0029 (3)	0.0557 (11)	
H3	1.1909	0.8014	0.0159	0.067*	
C4	1.0069 (4)	0.8582 (3)	0.0633 (3)	0.0500 (10)	
H4	1.0406	0.8702	0.1276	0.060*	
C5	0.8689 (4)	0.8814 (3)	0.0330 (3)	0.0384 (9)	
C6	0.7641 (4)	0.9318 (2)	0.0928 (3)	0.0369 (9)	
C7	0.6528 (4)	1.0085 (3)	0.2065 (3)	0.0394 (9)	
C8	0.6207 (4)	1.0541 (3)	0.3019 (2)	0.0553 (11)	
H8A	0.5369	1.0980	0.2930	0.066*	
H8B	0.7007	1.0988	0.3268	0.066*	
C9	0.5929 (5)	0.9754 (4)	0.3773 (3)	0.0924 (16)	
H9A	0.5175	0.9280	0.3523	0.139*	
H9B	0.5649	1.0117	0.4345	0.139*	
H9C	0.6789	0.9370	0.3926	0.139*	
C10	0.8771 (4)	0.9203 (3)	0.2639 (2)	0.0389 (9)	
C11	0.8735 (4)	0.8180 (3)	0.2893 (3)	0.0533 (11)	
H11	0.8095	0.7703	0.2565	0.064*	
C12	0.9657 (5)	0.7877 (3)	0.3639 (3)	0.0658 (13)	
H12	0.9645	0.7186	0.3816	0.079*	
C13	1.0597 (5)	0.8572 (4)	0.4129 (3)	0.0666 (13)	
H13	1.1212	0.8355	0.4639	0.080*	
C14	1.0629 (4)	0.9590 (4)	0.3866 (3)	0.0593 (12)	
H14	1.1270	1.0064	0.4196	0.071*	
C15	0.9716 (4)	0.9910 (3)	0.3117 (3)	0.0464 (10)	
H15	0.9737	1.0600	0.2936	0.056*	
C16	0.4266 (4)	0.6705 (3)	0.6369 (3)	0.0557 (11)	
H16	0.4034	0.6773	0.7006	0.067*	
C17	0.5618 (5)	0.7017 (3)	0.6151 (3)	0.0618 (12)	
H17	0.6275	0.7301	0.6630	0.074*	
C18	0.5970 (5)	0.6905 (3)	0.5229 (3)	0.0628 (12)	
H18	0.6884	0.7088	0.5070	0.075*	
C19	0.4956 (4)	0.6514 (3)	0.4526 (3)	0.0579 (12)	
H19	0.5170	0.6445	0.3885	0.069*	
C20	0.3628 (4)	0.6231 (3)	0.4788 (3)	0.0423 (10)	
C21	0.2472 (4)	0.5760 (2)	0.4143 (2)	0.0383 (9)	
C22	0.1065 (4)	0.5101 (3)	0.2944 (3)	0.0407 (10)	
C23	0.0445 (4)	0.4789 (3)	0.1960 (3)	0.0544 (11)	
H23A	−0.0399	0.4355	0.2013	0.065*	
H23B	0.1139	0.4367	0.1642	0.065*	
C24	0.0022 (5)	0.5691 (3)	0.1334 (3)	0.0805 (15)	
H24A	−0.0659	0.6120	0.1644	0.121*	
H24B	−0.0403	0.5424	0.0720	0.121*	
H24C	0.0860	0.6101	0.1239	0.121*	
C25	0.3299 (4)	0.5928 (3)	0.2453 (2)	0.0425 (10)	
C26	0.3472 (4)	0.6965 (3)	0.2290 (3)	0.0548 (11)	
H26	0.3004	0.7474	0.2641	0.066*	
C27	0.4356 (5)	0.7235 (3)	0.1596 (3)	0.0691 (13)	
H27	0.4487	0.7934	0.1469	0.083*	
C28	0.5050 (5)	0.6471 (4)	0.1087 (3)	0.0734 (14)	
H28	0.5646	0.6659	0.0617	0.088*	
C29	0.4870 (5)	0.5437 (4)	0.1266 (3)	0.0655 (13)	
H29	0.5344	0.4927	0.0921	0.079*	
C30	0.3985 (4)	0.5157 (3)	0.1956 (3)	0.0522 (11)	
H30	0.3854	0.4458	0.2085	0.063*	

### Atomic displacement parameters (Å^2^)

**Table:**

	*U*^11^	*U*^22^	*U*^33^	*U*^12^	*U*^13^	*U*^23^
Cu1	0.0498 (3)	0.0414 (3)	0.0382 (3)	0.0052 (2)	0.0051 (2)	−0.0032 (2)
Cu2	0.0533 (3)	0.0406 (3)	0.0373 (3)	−0.0008 (2)	0.0068 (2)	−0.0036 (2)
Cl1	0.1114 (10)	0.0409 (6)	0.0567 (7)	−0.0113 (6)	−0.0079 (7)	−0.0003 (5)
Cl2	0.0555 (7)	0.0697 (7)	0.0714 (8)	−0.0007 (6)	0.0146 (6)	0.0207 (6)
Cl3	0.0942 (9)	0.0391 (6)	0.0654 (8)	0.0070 (6)	0.0238 (7)	−0.0036 (5)
Cl4	0.0598 (7)	0.0617 (7)	0.0785 (8)	0.0086 (6)	0.0030 (6)	0.0178 (6)
N1	0.049 (2)	0.0439 (19)	0.040 (2)	0.0092 (15)	0.0050 (17)	−0.0072 (16)
N2	0.043 (2)	0.0409 (19)	0.0296 (18)	0.0014 (15)	0.0044 (16)	−0.0041 (14)
N3	0.045 (2)	0.0405 (19)	0.0354 (19)	−0.0031 (15)	0.0071 (16)	−0.0055 (15)
N4	0.042 (2)	0.0347 (18)	0.0361 (19)	0.0014 (15)	0.0034 (16)	−0.0010 (14)
N5	0.047 (2)	0.0438 (19)	0.0367 (19)	−0.0040 (15)	0.0073 (17)	−0.0075 (15)
N6	0.045 (2)	0.0377 (18)	0.0317 (18)	0.0015 (15)	0.0046 (16)	−0.0018 (14)
N7	0.045 (2)	0.0374 (18)	0.0343 (18)	−0.0021 (14)	0.0062 (16)	−0.0055 (15)
N8	0.055 (2)	0.0314 (17)	0.0344 (19)	0.0004 (15)	0.0110 (17)	−0.0014 (14)
C1	0.068 (3)	0.062 (3)	0.038 (2)	0.013 (2)	0.002 (2)	−0.009 (2)
C2	0.059 (3)	0.056 (3)	0.052 (3)	0.020 (2)	0.012 (2)	−0.004 (2)
C3	0.052 (3)	0.057 (3)	0.059 (3)	0.015 (2)	0.007 (2)	0.003 (2)
C4	0.052 (3)	0.061 (3)	0.037 (2)	0.004 (2)	0.005 (2)	0.000 (2)
C5	0.042 (3)	0.034 (2)	0.039 (2)	0.0020 (18)	0.004 (2)	−0.0001 (18)
C6	0.044 (3)	0.033 (2)	0.033 (2)	−0.0031 (18)	0.003 (2)	−0.0015 (18)
C7	0.042 (3)	0.041 (2)	0.035 (2)	−0.0011 (19)	0.003 (2)	0.0021 (18)
C8	0.052 (3)	0.074 (3)	0.038 (2)	0.012 (2)	0.004 (2)	−0.014 (2)
C9	0.096 (4)	0.141 (5)	0.044 (3)	0.022 (3)	0.022 (3)	0.018 (3)
C10	0.043 (2)	0.044 (2)	0.031 (2)	0.0070 (19)	0.0064 (19)	0.0028 (19)
C11	0.068 (3)	0.041 (3)	0.050 (3)	0.002 (2)	0.002 (2)	0.002 (2)
C12	0.083 (4)	0.049 (3)	0.068 (3)	0.018 (3)	0.012 (3)	0.017 (2)
C13	0.066 (3)	0.093 (4)	0.040 (3)	0.021 (3)	−0.004 (2)	0.015 (3)
C14	0.055 (3)	0.079 (3)	0.042 (3)	−0.002 (2)	−0.001 (2)	−0.007 (2)
C15	0.054 (3)	0.048 (2)	0.038 (2)	0.001 (2)	0.006 (2)	0.003 (2)
C16	0.062 (3)	0.064 (3)	0.041 (3)	−0.010 (2)	0.006 (2)	−0.009 (2)
C17	0.059 (3)	0.069 (3)	0.056 (3)	−0.014 (2)	−0.003 (2)	−0.005 (2)
C18	0.052 (3)	0.072 (3)	0.066 (3)	−0.014 (2)	0.012 (3)	0.001 (3)
C19	0.057 (3)	0.070 (3)	0.047 (3)	−0.006 (2)	0.009 (2)	−0.004 (2)
C20	0.048 (3)	0.037 (2)	0.043 (2)	0.0023 (19)	0.008 (2)	−0.0002 (18)
C21	0.049 (3)	0.032 (2)	0.034 (2)	0.0051 (18)	0.005 (2)	−0.0022 (17)
C22	0.053 (3)	0.031 (2)	0.039 (2)	0.0023 (18)	0.009 (2)	−0.0044 (18)
C23	0.062 (3)	0.059 (3)	0.041 (3)	−0.006 (2)	0.010 (2)	−0.012 (2)
C24	0.107 (4)	0.090 (4)	0.043 (3)	0.000 (3)	−0.008 (3)	0.015 (3)
C25	0.053 (3)	0.044 (2)	0.032 (2)	0.001 (2)	0.0100 (19)	0.0029 (18)
C26	0.067 (3)	0.041 (3)	0.059 (3)	0.000 (2)	0.012 (2)	0.006 (2)
C27	0.077 (4)	0.062 (3)	0.070 (3)	−0.006 (3)	0.007 (3)	0.024 (3)
C28	0.070 (4)	0.108 (4)	0.044 (3)	−0.005 (3)	0.013 (3)	0.018 (3)
C29	0.073 (3)	0.081 (4)	0.044 (3)	0.012 (3)	0.018 (2)	−0.002 (2)
C30	0.069 (3)	0.050 (3)	0.039 (2)	0.007 (2)	0.013 (2)	0.001 (2)

### Geometric parameters (Å, °)

**Table:**

Cu1—N3^i^	1.989 (3)	C9—H9A	0.9600
Cu1—N1	2.029 (3)	C9—H9B	0.9600
Cu1—N2	2.179 (3)	C9—H9C	0.9600
Cu1—Cl2	2.2657 (11)	C10—C15	1.372 (5)
Cu1—Cl1	2.2734 (10)	C10—C11	1.374 (4)
Cu2—N7^ii^	1.977 (3)	C11—C12	1.367 (5)
Cu2—N5	2.018 (3)	C11—H11	0.9300
Cu2—N6	2.181 (3)	C12—C13	1.366 (5)
Cu2—Cl4	2.2637 (12)	C12—H12	0.9300
Cu2—Cl3	2.2804 (11)	C13—C14	1.371 (5)
N1—C1	1.328 (4)	C13—H13	0.9300
N1—C5	1.340 (4)	C14—C15	1.373 (5)
N2—C6	1.302 (4)	C14—H14	0.9300
N2—N3	1.382 (3)	C15—H15	0.9300
N3—C7	1.311 (4)	C16—C17	1.382 (5)
N3—Cu1^i^	1.989 (3)	C16—H16	0.9300
N4—C6	1.362 (4)	C17—C18	1.352 (5)
N4—C7	1.365 (4)	C17—H17	0.9300
N4—C10	1.438 (4)	C18—C19	1.380 (5)
N5—C16	1.322 (4)	C18—H18	0.9300
N5—C20	1.342 (4)	C19—C20	1.371 (5)
N6—C21	1.309 (4)	C19—H19	0.9300
N6—N7	1.371 (3)	C20—C21	1.457 (5)
N7—C22	1.317 (4)	C22—C23	1.480 (5)
N7—Cu2^ii^	1.977 (3)	C23—C24	1.515 (5)
N8—C22	1.358 (4)	C23—H23A	0.9700
N8—C21	1.364 (4)	C23—H23B	0.9700
N8—C25	1.457 (4)	C24—H24A	0.9600
C1—C2	1.374 (5)	C24—H24B	0.9600
C1—H1	0.9300	C24—H24C	0.9600
C2—C3	1.366 (5)	C25—C26	1.367 (4)
C2—H2	0.9300	C25—C30	1.373 (4)
C3—C4	1.382 (4)	C26—C27	1.374 (5)
C3—H3	0.9300	C26—H26	0.9300
C4—C5	1.362 (5)	C27—C28	1.379 (5)
C4—H4	0.9300	C27—H27	0.9300
C5—C6	1.471 (4)	C28—C29	1.369 (5)
C7—C8	1.482 (4)	C28—H28	0.9300
C8—C9	1.522 (5)	C29—C30	1.375 (5)
C8—H8A	0.9700	C29—H29	0.9300
C8—H8B	0.9700	C30—H30	0.9300
			
N3^i^—Cu1—N1	172.45 (11)	H9A—C9—H9B	109.5
N3^i^—Cu1—N2	95.70 (10)	C8—C9—H9C	109.5
N1—Cu1—N2	77.15 (11)	H9A—C9—H9C	109.5
N3^i^—Cu1—Cl2	91.19 (9)	H9B—C9—H9C	109.5
N1—Cu1—Cl2	89.93 (9)	C15—C10—C11	121.0 (4)
N2—Cu1—Cl2	116.78 (8)	C15—C10—N4	119.9 (3)
N3^i^—Cu1—Cl1	92.67 (8)	C11—C10—N4	118.9 (3)
N1—Cu1—Cl1	92.30 (8)	C12—C11—C10	118.6 (4)
N2—Cu1—Cl1	112.17 (8)	C12—C11—H11	120.7
Cl2—Cu1—Cl1	130.20 (4)	C10—C11—H11	120.7
N7^ii^—Cu2—N5	173.36 (11)	C13—C12—C11	121.2 (4)
N7^ii^—Cu2—N6	96.47 (10)	C13—C12—H12	119.4
N5—Cu2—N6	77.15 (11)	C11—C12—H12	119.4
N7^ii^—Cu2—Cl4	90.06 (9)	C12—C13—C14	119.7 (4)
N5—Cu2—Cl4	90.60 (9)	C12—C13—H13	120.2
N6—Cu2—Cl4	111.51 (8)	C14—C13—H13	120.2
N7^ii^—Cu2—Cl3	91.26 (8)	C13—C14—C15	120.1 (4)
N5—Cu2—Cl3	92.95 (8)	C13—C14—H14	119.9
N6—Cu2—Cl3	113.03 (8)	C15—C14—H14	119.9
Cl4—Cu2—Cl3	134.97 (4)	C10—C15—C14	119.3 (4)
C1—N1—C5	117.9 (3)	C10—C15—H15	120.3
C1—N1—Cu1	122.4 (3)	C14—C15—H15	120.3
C5—N1—Cu1	119.3 (2)	N5—C16—C17	122.5 (4)
C6—N2—N3	106.7 (3)	N5—C16—H16	118.8
C6—N2—Cu1	111.1 (2)	C17—C16—H16	118.8
N3—N2—Cu1	141.9 (2)	C18—C17—C16	119.0 (4)
C7—N3—N2	108.3 (3)	C18—C17—H17	120.5
C7—N3—Cu1^i^	129.5 (2)	C16—C17—H17	120.5
N2—N3—Cu1^i^	122.2 (2)	C17—C18—C19	119.2 (4)
C6—N4—C7	105.2 (3)	C17—C18—H18	120.4
C6—N4—C10	130.0 (3)	C19—C18—H18	120.4
C7—N4—C10	124.5 (3)	C20—C19—C18	118.8 (4)
C16—N5—C20	118.4 (3)	C20—C19—H19	120.6
C16—N5—Cu2	122.0 (3)	C18—C19—H19	120.6
C20—N5—Cu2	119.4 (2)	N5—C20—C19	122.0 (3)
C21—N6—N7	107.3 (3)	N5—C20—C21	112.5 (3)
C21—N6—Cu2	111.1 (2)	C19—C20—C21	125.4 (3)
N7—N6—Cu2	141.4 (2)	N6—C21—N8	109.6 (3)
C22—N7—N6	108.4 (3)	N6—C21—C20	119.5 (3)
C22—N7—Cu2^ii^	129.7 (3)	N8—C21—C20	130.9 (3)
N6—N7—Cu2^ii^	122.0 (2)	N7—C22—N8	108.7 (3)
C22—N8—C21	106.1 (3)	N7—C22—C23	125.6 (3)
C22—N8—C25	124.3 (3)	N8—C22—C23	125.7 (3)
C21—N8—C25	129.6 (3)	C22—C23—C24	114.7 (3)
N1—C1—C2	123.0 (4)	C22—C23—H23A	108.6
N1—C1—H1	118.5	C24—C23—H23A	108.6
C2—C1—H1	118.5	C22—C23—H23B	108.6
C3—C2—C1	118.6 (4)	C24—C23—H23B	108.6
C3—C2—H2	120.7	H23A—C23—H23B	107.6
C1—C2—H2	120.7	C23—C24—H24A	109.5
C2—C3—C4	119.0 (4)	C23—C24—H24B	109.5
C2—C3—H3	120.5	H24A—C24—H24B	109.5
C4—C3—H3	120.5	C23—C24—H24C	109.5
C5—C4—C3	118.9 (4)	H24A—C24—H24C	109.5
C5—C4—H4	120.5	H24B—C24—H24C	109.5
C3—C4—H4	120.5	C26—C25—C30	122.4 (4)
N1—C5—C4	122.5 (3)	C26—C25—N8	119.8 (3)
N1—C5—C6	111.9 (3)	C30—C25—N8	117.8 (3)
C4—C5—C6	125.5 (3)	C25—C26—C27	118.3 (4)
N2—C6—N4	110.7 (3)	C25—C26—H26	120.9
N2—C6—C5	119.8 (3)	C27—C26—H26	120.9
N4—C6—C5	129.3 (4)	C26—C27—C28	120.2 (4)
N3—C7—N4	109.1 (3)	C26—C27—H27	119.9
N3—C7—C8	126.4 (4)	C28—C27—H27	119.9
N4—C7—C8	124.4 (3)	C29—C28—C27	120.6 (4)
C7—C8—C9	115.4 (3)	C29—C28—H28	119.7
C7—C8—H8A	108.4	C27—C28—H28	119.7
C9—C8—H8A	108.4	C28—C29—C30	119.8 (4)
C7—C8—H8B	108.4	C28—C29—H29	120.1
C9—C8—H8B	108.4	C30—C29—H29	120.1
H8A—C8—H8B	107.5	C25—C30—C29	118.8 (4)
C8—C9—H9A	109.5	C25—C30—H30	120.6
C8—C9—H9B	109.5	C29—C30—H30	120.6

Symmetry codes: (i) −*x*+1, −*y*+2, −*z*; (ii) −*x*, −*y*+1, −*z*+1.

### Hydrogen-bond geometry (Å, °)

**Table:**

*D*—H···*A*	*D*—H	H···*A*	*D*···*A*	*D*—H···*A*
C4—H4···Cl2^iii^	0.93	2.78	3.445 (4)	129
C15—H15···Cl3^iv^	0.93	2.78	3.579 (4)	145
C16—H16···Cl1^v^	0.93	2.76	3.518 (4)	139
C19—H19···Cl4^vi^	0.93	2.69	3.365 (4)	130

Symmetry codes: (iii) −*x*+2, −*y*+2, −*z*; (iv) −*x*+1, −*y*+2, −*z*+1; (v) *x*, *y*, *z*+1; (vi) −*x*+1, −*y*+1, −*z*+1.
